# Predicting the risk of locoregional recurrence after early breast cancer: an external validation of the Dutch INFLUENCE-nomogram with clinical cancer registry data from Germany

**DOI:** 10.1007/s00432-019-02904-4

**Published:** 2019-03-29

**Authors:** Vinzenz Voelkel, Teresa Draeger, Catharina G. M. Groothuis-Oudshoorn, Linda de Munck, Tom Hueting, Michael Gerken, Monika Klinkhammer-Schalke, Miha Lavric, Sabine Siesling

**Affiliations:** 1 Institute for Quality Control and Health Services Research, Tumor Center Regensburg/University of Regensburg, Regensburg, Germany; 2 Department of Health Technology and Services Research, Technical Medical Centre, University of Twente, Enschede, The Netherlands; 3 Evidencio, Medical Decision Support, Haaksbergen, The Netherlands; 4 Department of Research, Netherlands Comprehensive Cancer Organisation (IKNL), Utrecht, The Netherlands

**Keywords:** Mamma carcinoma, Personalized care, Follow-up, Tertiary prevention, Cancer registry, Health services research

## Abstract

**Purpose:**

Follow-up after breast cancer treatment aims for an early detection of locoregional breast cancer recurrences (LRR) to improve the patients’ outcome. By estimating individual’s 5-year recurrence-risks, the Dutch INFLUENCE-nomogram can assist health professionals and patients in developing personalized risk-based follow-up pathways. The objective of this study is to validate the prediction tool on non-Dutch patients.

**Material and methods:**

Data for this external validation derive from a large clinical cancer registry in southern Germany, covering a population of 1.1 million. Patients with curative resection of early-stage breast cancer, diagnosed between 2000 and 2012, were included in the analysis (*n = *6520). For each of them, an individual LRR-risk was estimated by the INFLUENCE-nomogram. Its predictive ability was tested by comparing estimated and observed LRR-probabilities using the Hosmer–Lemeshow goodness-of-fit test and C-statistics.

**Results:**

In the German validation-cohort, 2.8% of the patients developed an LRR within 5 years after primary surgery (*n = *184). While the INFLUENCE-nomogram generally underestimates the actual LRR-risk of the German patients (*p < *0.001), its discriminative ability is comparable to the one observed in the original Dutch modeling-cohort (C-statistic German validation-cohort: 0.73, CI 0.69–0.77 vs. C-statistic Dutch modeling-cohort: 0.71, CI 0.69–0.73). Similar results were obtained in most of the subgroup analyses stratified by age, type of surgery and intrinsic biological subtypes.

**Conclusion:**

The outcomes of this external validation underline the generalizability of the INFLUENCE-nomogram beyond the Dutch population. The model performance could be enhanced in future by incorporating additional risk factors for LRR.

## Purpose

Breast cancer is the most frequent malignancy among the female population. Worldwide, approximately 1.7 million women per year are diagnosed with this kind of tumor (Stewart and Wild 2014). Due to early detection, leading to lower stage at diagnosis, better treatment strategies and a wider awareness for the disease in general, the survival rates of breast cancer patients have been increasing considerably during the past decades (Yoshimura et al. 2018; Holleczek et al. 2011; Holleczek and Brenner 2012). In early-stage breast cancer, radical removal of the tumor is the first choice of treatment, which is often followed by adjuvant radiation and/or systemic therapy. Additionally, tertiary prevention is of great importance. Patients undergo regular follow-up visits to detect possible locoregional recurrences (LRR) in an early stage. Thus, subsequent distant metastases with a high risk of poor outcome shall be avoided (Lu et al. 2009; Sangen et al. 2013). Fortunately, the LRR-rate is generally low. Only 4% of all Dutch breast cancer patients from the year 2003 were diagnosed with a local recurrence event within 10 years after primary surgery, and only 2% developed a regional recurrence (Geurts et al. 2017). Moreover, the risk of LRR is not the same for every patient and changes over time. Since every follow-up visit potentially affects a patient’s quality of life (Puglisi et al. 2014) and is a burden on health care facilities, it might be reasonable to personalize follow-up schemes by focusing on high-risk patients. In 2015, Witteveen et al. developed a “prognostic nomogram for the estimation of annual risk of locoregional recurrence in early breast cancer patients” (Witteveen et al. 2015). This so-called INFLUENCE-nomogram is based on over 37,000 patients of the Netherlands cancer registry (NCR) from the years 2003–2006. After entering several patient, tumor, and treatment characteristics (age, tumor size, nodal involvement, grade, ER-/PR-status, multifocality, radiotherapy, chemotherapy, and endocrine therapy), it estimates the individual risk of developing a recurrence within the first 5 years after surgery, as well as conditional annual risks based on multivariable logistic regression models (Witteveen 2019; Witteveen 2015). To assess the validity of this online tool, it was tested successfully on another Dutch cohort of more than 12,000 patients from the years 2007–2008 (Witteveen et al. 2015). However, until today it is unclear whether the nomogram is generalizable to foreign populations and health care systems, which would contribute to demonstrate its clinical relevance. This study aims to test the external validity of the INFLUENCE-nomogram on a representative cohort from a large German clinical cancer registry with additional emphasis on important patient subgroups.

## Methods

Data for this external validation derive from a clinical cancer registry (Tumor Center Regensburg/University of Regensburg, Institute for Quality Control and Health Services Research (Tumorzentrum Regensburg 2019), which systematically collects medical records of all tumor patients registered within a large political district in the south of Germany compromising approximately 1.1 million inhabitants (Bayerisches Landesamt für Statistik und Datenverarbeitung 2014). Information on each patient includes demographics, tumor characteristics, surgical procedure, and adjuvant treatment (Table 1). Over 50 hospitals and 1500 registered health professionals immediately report newly diagnosed recurrent events. To obtain actual information on vital status, a regular exchange with local registration and public health offices takes place. All following analyses are performed in compliance with German data protection laws.

**Table 1:** A patient and tumor characteristics; B treatment characteristics

	Validation-cohort Germany (2000–2012) *n = *6520				Modeling-cohort Netherlands (2003–2006) *n = *37,278		
	*n*		%*		*n*		%*
(A) Patients and tumor characteristics							
Age category at diagnosis (years)							
< 50	1408		21.6		9779		26.2
50–59	1579		24.2		10,601		28.4
60–69	1866		28.6		8421		22.6
≥ 70	1667		25.6		8477		22.7
Histologic type							
Ductal	4812		73.8		29,582		79.4
Lobular	809		12.4		4000		10.7
Mixed	322		4.9		1552		4.2
Other	577		8.8		2144		5.8
Grading							
1	996		15.3		7628		22.0
2	3712		56.9		15,595		44.9
3	1812		27.8		11,479		33.1
Unknown	n.a.				2576		
Tumor size (mm)							
< 20	3699		56.7		22,611		61.2
20–50	2572		39.4		13,243		35.8
> 50	249		3.8		1094		3.0
Unknown	n.a.				330		
Multifocal							
No	5432		83.3		23,237		84.8
Yes	1088		16.7		4168		15.2
Unknown	n.a.				9873		
Lymph node status							
Negative	4660		71.5		22,516		61.3
1–3 positive	1079		16.5		10,093		27.5
> 3 positive	781		12.0		4119		11.2
Unknown	n.a.				550		
ER status							
Negative	982		15.1		5417		18.8
Positive	5538		84.9		23,433		81.2
Unknown	n.a.				8428		
PR status							
Negative	1487		22.8		9580		33.7
Positive	5033		77.2		18,877		66.3
Unknown	n.a.				8821		
Her2neu status^+^							
Negative	4850		81.9		13,832		85.2
Positive	1074		18.1		2405		14.8
Unknown	596				21,041		
Intrinsic biological subtype							
Luminal A/B	5661		88.0		n.a.		
Her2neu positive	249		3.9				
Triple negative	521		8.1				
Unknown	89						
(B) Treatment characteristics							
Type of surgery							
Breast conserving	4695		72.0		21,049		56.5
Mastectomy	1722		26.4		16,229		43.5
Unknown	103				n.a.		
Chemotherapy							
No	3518		54.0		23,886		64.1
Yes	3002		46.0		13,392		35.9
Radiotherapy							
No	1573		24.1		12,783		34.3
Yes	4947		75.9		24,495		65.7
Endocrine therapy							
No	1501		23.0		21,696		58.2
Yes	5019		77.0		15,582		41.8

+ Her2neu was determined routinely after the introduction of Trastuzumab antibody-therapy in 2005

*Percentages do not consider patients with unknown variable values

To be included in the validation process, patients had to fulfill certain inclusion criteria dictated by the INFLUENCE-nomogram. All patients with a histologically confirmed primary invasive breast cancer diagnosis (ICD-10-GM C50 (DIMDI 2017) between 2000 and 2012, who according to their OPS-code (Institut 2019) received curative R0-resection (Hermanek and Wittekind 1994) were eligible for inclusion. Patients who received neoadjuvant treatment were diagnosed with distant metastasis or T4-tumors were excluded for analysis. Of all patients meeting the inclusion criteria, only patients without missing data on any relevant item and a follow-up time of at least 5 years were used for validation of the 5-year overall risk predictions. To assess the separate predictions for the five conditional annual risks, patients were required to have a minimum follow-up time of 1, 2, 3, 4 and 5 years, respectively. To account for selection bias due to exclusion, a sensitivity analysis comparing LRR-rates was performed.

Patient, tumor and treatment characteristics of the German validation-cohort were compared to those of the Dutch cohort from 2003 to 2006, on which the prediction tool was originally built (hereafter referred to as “modeling-cohort”) (Witteveen et al. 2015). For this purpose, it was refrained from using χ-square tests, since with large numbers of observation units, they are overly sensitive to minor, and from a clinical point of view irrelevant, differences in distribution.

To obtain the German validation-cohort’s interval-specific LRR-rates, the life-table method based on five annual observation periods was employed. Thereafter each patient’s individual recurrence risk was estimated using the algorithm behind the INFLUENCE-nomogram (obtained from the online medical prediction platform Evidencio, www.evidencio.com (Witteveen 2019) both for the whole 5-year postoperative period and for every year separately. To assess general prediction accuracy on the overall 5-year LRR-risk, the Hosmer–Lemeshow goodness-of-fit test based on quintiles was employed (Hosmer et al. 2013). Confidence intervals for the observed LRR-rates were obtained using Clopper–Pearson’s exact method based on binomial distribution (Clopper and Pearson 1934). Moreover, a calibration chart was plotted to visualize the correlation between all predicted probabilities and the observed primary LRR-rates. Due to a low annual number of primary LRRs in the validation-cohort, it was refrained from performing separate Hosmer–Lemeshow tests for every year.

The ultimate aim of prediction tools like the INFLUENCE-nomogram is to discern between high- and low-risk patients (Steyerberg et al. 2010). This discrimination-ability can be evaluated using the C-statistic/ area under the curve (AUC) of the receiver-operator characteristic (ROC) (Bamber 1975). A C-statistic (AUC) of 1.0 indicates perfect predictive ability, whereas 0.5 represents no predictive discrimination. The corresponding confidence intervals were obtained by DeLong’s method (DeLong et al. 1988). Based on the ROC curve of the German validation-cohort, the model’s joint-optimum for sensitivity and specificity based on an “ideal” cutoff risk value was calculated using Youden’s J-statistic (Youden 1950). With nonparametric estimation, ROC actually requires fewer distributional assumptions than the Hosmer–Lemeshow test (Youngstrom 2013). Thus, it is not only possible to validate the discrimination-ability of the 5-year overall predictions but also of the time-dependent model estimating annual risks (conditional on the fact that a patient did not develop a recurrence in the previous year). Additionally to that, analyses stratified by age, type of surgery, and intrinsic biological subgroups (following the definition of the 12th St. Gallen International Breast Cancer Conference, 2011 (Goldhirsch et al. 2011) could be performed.

All significance tests were two-sided with a significance level of 0.05 and results are displayed with 95% confidence intervals (CI). The findings of this survey are presented in strict compliance with the Strengthening the Reporting of Observational studies in Epidemiology (STROBE) statement (Elm et al. 2007). During this study, IBM SPSS 25 (IBM Corp., SPSS for Windows, Armonk, NY, USA), as well as R version 3.3.2 (R Foundation for Statistical Computing, Vienna, Austria; https://www.R-project.org/) and the R packages “predictABEL” (Kundu et al. 2011), “Hmisc” (www.CRAN.R-project.org/package=Hmisc) and “pROC” (Robin et al. 2011) were used.

## Results

**Fig. 1:**
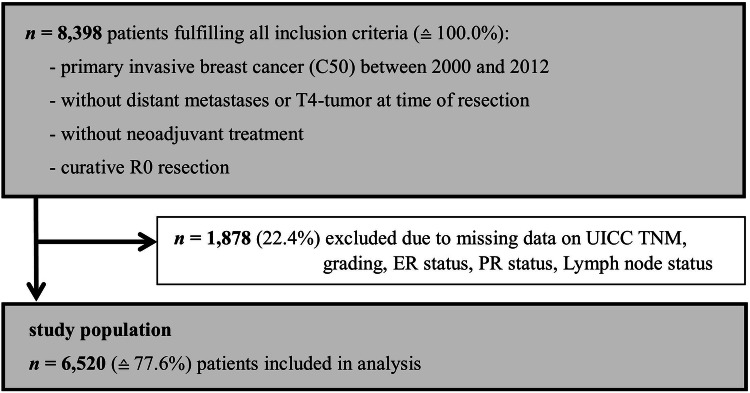
Flowchart of study patient selection

Within the observed German political district, 8398 patients diagnosed with invasive breast cancer between 2000 and 2012 fulfilled all inclusion criteria dictated by the INFLUENCE-nomogram (no distant metastases or T4-stage at time of surgery, no neoadjuvant therapy, no micro- or macroscopically incomplete surgery). Of them, 1878 (22.4%) had to be excluded due to missing data on UICC TNM, grading, hormone receptor or lymph node status, leaving 6520 patients that could be used for validation (Fig. 1).

The median (mean) follow-up time calculated over the whole validation-cohort was 8.5 (8.6) years (using Korn’s Kaplan–Meier potential follow-up method (Schemper and Smith 1996). One hundred and eighty-four patients developed an LRR within 5 years, which is equivalent to an overall 5-year LRR-rate of 2.8%. This percentage is not significantly different to the LRR-rate observed in the Dutch modeling-cohort (2.6%, *p = *0.205). The LRR-rate among the 1878 excluded patients was 2.9%, which, according to the sensitivity analysis, is not significantly different from the included patients’ LRR-rate (*p = *0.902). Thus, selection bias due to exclusion seems unlikely.

**Fig. 2:**
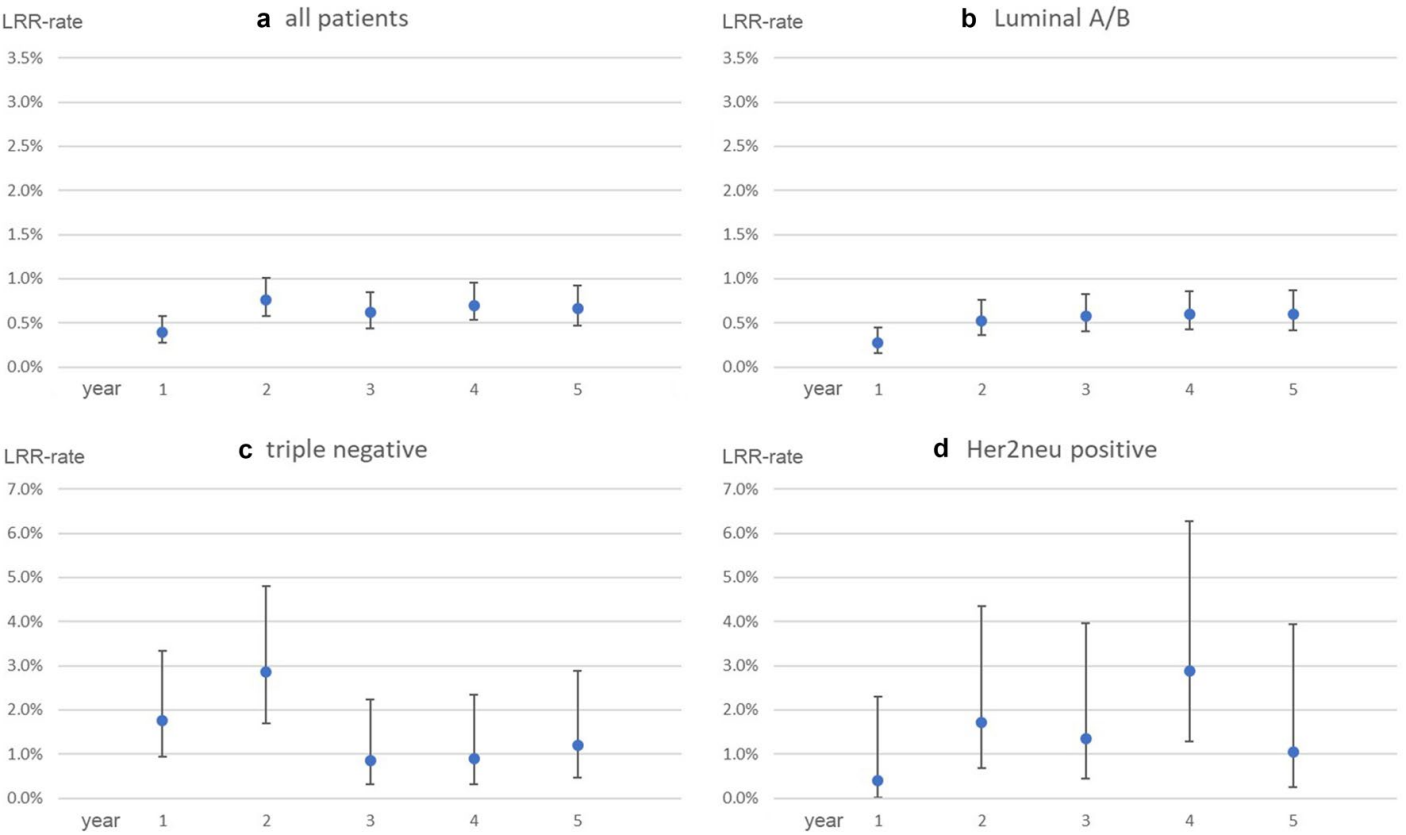
Annual risks for LRR observed in the validation-cohort with 95% CI. **a** All patients. **b** Luminal A/B. **c** Triple negative. **d** Her2neu positive

The observed LRR-rates in the German validation-cohort range between 0.4% and 0.8% per year (Fig. 2a). After stratifying for intrinsic biological subtypes, a similar trend can be seen among Luminal A/B patients (Fig. 2b). Triple-negative patients show an incidence-peak in the second year after surgery (LRR-rate year 2: 2.9%, Fig. 2c). With Her2neu-positive patients, the LRR-rate reaches its maximum in year 4 (LRR-rate year 4: 3.0%), although no clear trend can be identified (Fig. 2d). However, this subgroup is formed by 249 patients, which goes along with a small number of LRR-events. Consequently, the confidence intervals for the Her2neu patients’ LRR-rate are quite large (e.g. year 4: CI 1.4%–6.4%).

The German validation- and the Dutch modeling-cohort exhibit highly comparable patient and tumor features. Generally, there are low absolute differences in relative proportions of variables, e.g. age category at diagnosis, tumor size, or estrogen receptor (ER)-status (Table 1a). More substantial discrepancies between the Dutch and the German patients can be observed among the treatment variables (Table 1b). While 72% of the German patients received breast-conserving surgery, just 57% of the Dutch patients were treated this way. Additionally, there was a higher rate of endocrine therapy to be observed in the German cohort, although hormone status did not differ substantially between groups. Adjuvant chemo- or radiotherapy was performed more often in Germany, too.

## Validation

**Fig. 3:**
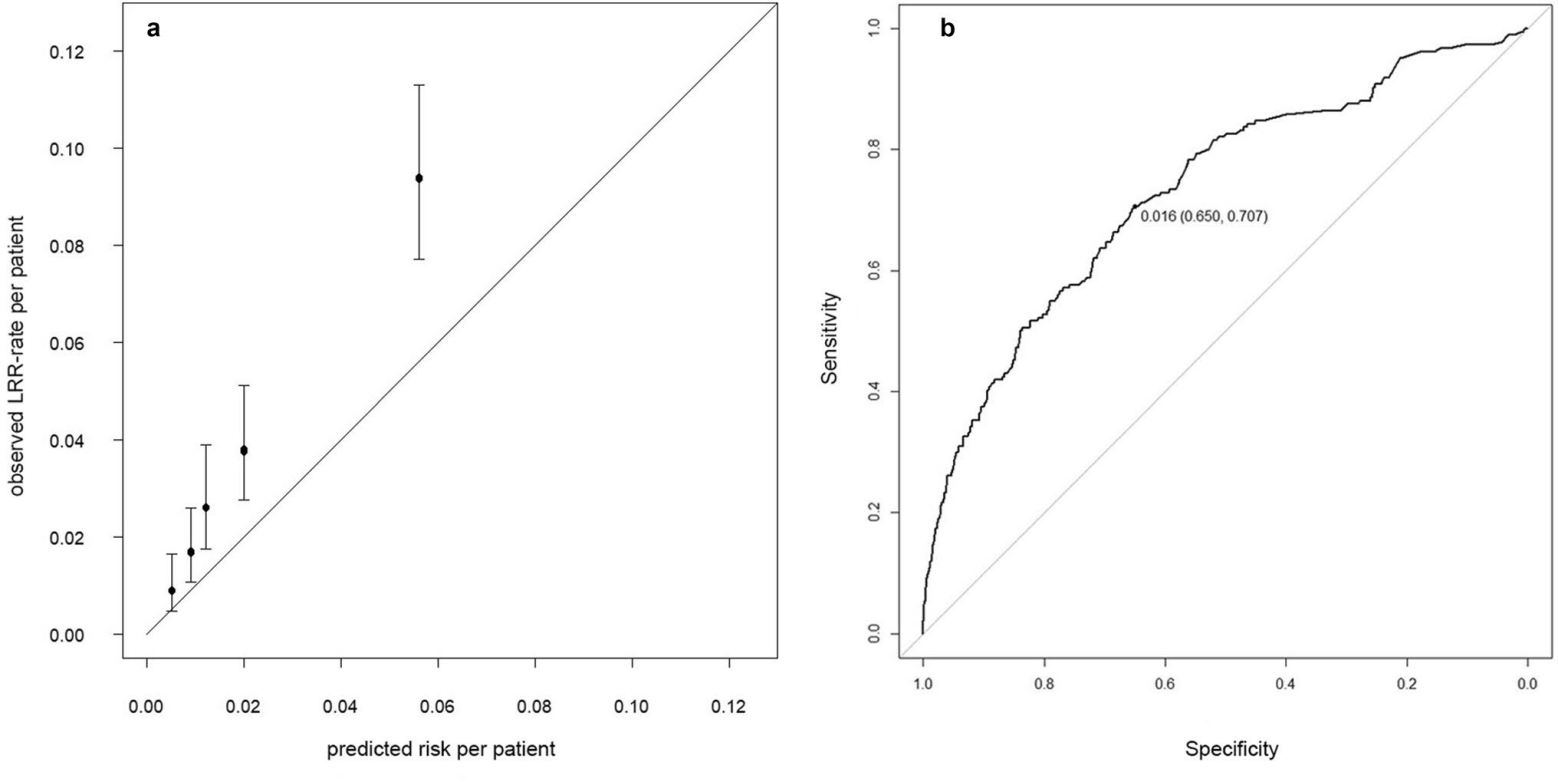
5-year overall LRR-risk. **a** Calibration chart of the validation-cohort based on quintiles. Hosmer–Lemeshow goodness-of-fit test: *p < *0.001. **b** 5-year overall LRR-risk. ROC curve of the validation-cohort (C-statistic/AUC: 0.73, CI 0.69–0.77) together with the optimal discrimination-threshold according to Youden’s J-statistic: cutoff value (specificity, sensitivity)

Initially, the 5-year LRR-risks estimated by the INFLUENCE-nomogram were compared to the actually observed recurrence rates within the validation-cohort using the Hosmer–Lemeshow goodness-of-fit test, which returned a *p* value lower than 0.001, indicating poor accuracy. However, looking at the calibration chart it becomes obvious that the absolute differences between observed and predicted risks are only moderate and come along with relatively large confidence intervals (Fig. 3a). Notwithstanding that, the INFLUENCE-nomogram tends to underestimate the 5-year LRR-risk. In the quintile comprising the patients with the lowest risk estimations, a mean predicted LRR-risk of 0.5% stands against a mean observed LRR-rate of 0.9% (CI 0.5–1.6%). In the highest risk quintile, the difference between the mean predicted LRR-risk and the mean observed LRR-rate is even significant (mean predicted: 5.6% vs. mean observed: 9.4%, CI 7.7–11.3%).

**Fig. 4:**
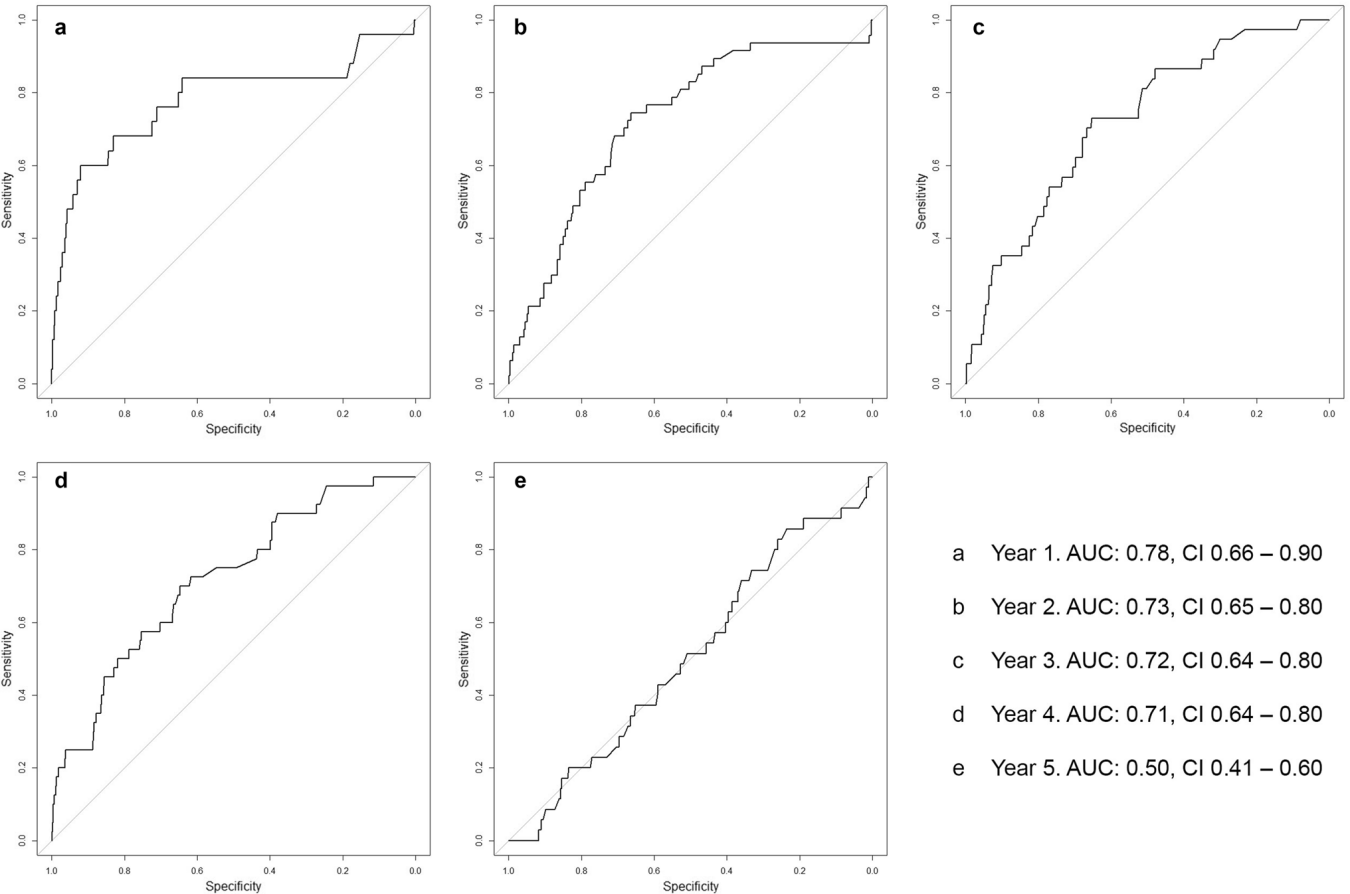
Annual LRR-risk: ROC curves of the validation-cohort

The INFLUENCE-nomogram’s discrimination-ability was evaluated by computing the C-statistic/area under the ROC curve, which was 0.73 (CI 0.69–0.77), indicating reasonably good performance (Fig. 3b). Given the according to Youden’s J-statistic ideal threshold of 1.6%, the joint-maximum of sensitivity and specificity is 70.7% and 65.0%, respectively. The discrimination-ability after predicting risks for each year separately is decreasing over time. While the C-statistic (AUC) is 0.78 (CI 0.66–0.90) for year 1 and 0.73 (CI 0.65–0.80) for year 2, it decreases to 0.50 (CI 0.41–0.60) in year 5, meaning there is no discriminative ability left (Fig. 4a–e).

The discriminative ability based on LRR-risk estimations for the whole 5-year period was tested for each of the age categories separately. The C-statistic (AUC) varied between 0.69 (CI 0.61–0.78) for patients younger than 50 years and 0.75 (CI 0.68–0.82) for patients older than 70 years (Table 2). Therefore, age is not an effect modifier for model performance. The same is true for the type of surgery (Table 2): the C-statistic is almost identical for patients with breast-conserving therapy (C-statistic/AUC 0.72, CI 0.67–0.78) and mastectomy (C-statistic/AUC 0.72, CI 0.65–0.78). Finally, another stratified analysis was performed for intrinsic biological subtypes (Table 2). For luminal A/B-tumors and triple-negative tumors, the C-statistic/AUC was 0.71 (CI 0.66–0.76) and 0.73 (CI 0.64–0.82), respectively. For Her2neu-positive patients, the model performed considerably worse (C-statistic/AUC 0.60, CI 0.43–0.76).

**Table 2:** Evaluation of discriminative ability by subgroups

Subgroups	AUC (C-statistic)	CI (lower–upper)
Age category at diagnosis (years)		
< 50	0.69	0.61–0.78
50–59	0.75	0.66–0.83
60–69	0.71	0.62–0.81
≥ 70	0.75	0.68–0.82
Type of surgery		
Breast conserving	0.72	0.67–0.78
Mastectomy	0.72	0.65–0.78
Intrinsic biological subtype		
Luminal A/B	0.71	0.66–0.76
Her2neu positive	0.60	0.43–0.76
Triple negative	0.73	0.64–0.82

## Discussion

An essential step towards the implementation of prediction tools in daily clinical practice is the validation in the target population (Steyerberg 2009). The present study is the first one testing the Dutch INFLUENCE-nomogram with external data from another country. Although its predictions for the LRR-risk in the German cohort comprising 6520 breast cancer patients were less accurate than in the Dutch modeling-cohort, it did not perform worse in terms of discrimination-ability (C-statistic/AUC German validation-cohort: 0.73, CI 0.69–0.77 vs. C-statistic/AUC Dutch modeling-cohort: 0.71, CI 0.69–0.73).

Germany and the Netherlands are direct European neighbors which have many things in common. Both the Netherlands Cancer Registry and the German Tumor Center Regensburg (as part of the Population based Cancer Registry Bavaria) are member of the European Network of Cancer Registries (https://www.encr.eu/) and follow the mandatory data-collection standards and dataset requirements developed by this network. But the similarities between the two countries go beyond registration rules. Similarities are also reflected by highly similar patient and tumor characteristics. Moreover, the national breast cancer treatment guidelines of the Netherlands and Germany exhibit a large degree of congruency, since they rest on the same evidence base like in many countries (NABON 2012; Leitlinienprogramm Onkologie (Deutsche Krebsgesellschaft, Deutsche Krebshilfe, AWMF) 2019; Wolters et al. 2012). Nevertheless, some substantial differences concerning treatment modalities can be observed, pointing to different national preferences in breast cancer treatment. As a matter of fact, the breast-conserving surgery rate in the Dutch cohort is 21.5% lower than in the German cohort, which also might explain the less frequent use of adjuvant radiotherapies. There are several potential reasons for this difference. First of all, the Dutch cohort derives from the years 2003 to 2006, whereas half of the German patients were treated thereafter. Between 2000 and 2012, the rate of breast-conserving surgery in the Netherlands progressed from 54 to 72% (Maaren et al. 2018). Second, one has to bear in mind that the Dutch patients as a whole are compared to a single region in Germany. Even in a small country like the Netherlands, large interregional variation exists concerning the use of breast-conserving surgery. According to a recent publication of van Maaren et al. (Maaren et al. 2018), some Dutch regions featured breast-conserving surgery rates slightly below 80% already in the last decade, while others did not reach the 60% threshold as late as 2015. Variation only decreased slightly after adjusting for different case mixes. It is very likely that a similar variation can be observed in Germany. The hospitals in the southern German region that we used for validation very actively participate in scientific research, which explains their early and broad implementation of the breast-conserving approach. However, the different national preferences concerning the surgical approach should not have influenced the results of our study, since the LRR-rate is comparable between breast-conserving surgery and mastectomy (Yang et al. 2008). Moreover, type of surgery does not contribute directly to the predictions of the INFLUENCE-nomogram, since breast-conserving surgery was strongly related to radiation therapy and, therefore, only the latter variable was included in the model (Witteveen et al. 2015).

For the considerably lower rate of endocrine therapy in the Netherlands, there might be another explication. The hormone status was unknown for over 20% of the Dutch patients, presumably because no tests were performed. Consequently, these patients were not eligible for hormone therapy. However, still only two-thirds of the patients with known hormone status received hormone therapy compared to around 90% in the German cohort.

Regardless of such differences, the LRR-rate was comparable between both countries and it seems justified to use the German cohort for external validation. Even if therapy-allocation in both cohorts is different to a certain degree, the same surgical techniques, drugs for hormonal- and chemotherapy and radiation-schemes are used (NABON 2012; Leitlinienprogramm Onkologie (Deutsche Krebsgesellschaft, Deutsche Krebshilfe, AWMF) 2019). With a total of 184 recurrence events, it also meets an important formal requirement for an external validation, as according to Vergouwe et al. at least 100 events and 100 “nonevents” are necessary to determine whether a prediction tool performs well or not (Vergouwe et al. 2005). The rate of 2.8% LRR in the German validation-cohort is mildly, but not significantly (p = 0.205) above the level in the Dutch modeling-cohort (2.6%). Recently, van Maaren et al. published a paper reviewing long-term recurrence rates for breast cancer based on comprehensive NCR data from 2005 showing the hazard on LRR-events of Her2neu-positive and triple-negative patients peaks within the second post-surgical year and drops thereafter (Maaren et al. 2018). No clear trends were seen in Luminal A or B patients. The findings concerning the three latter groups could be confirmed within the German validation-cohort. No clear trend was to be seen with the Her2neu-positive patients. One reason for that might be the small number of patients within this group, which is also reflected by large confidence intervals—one recurrence event more or less can already change the situation considerably. Another possible reason for these differing observations are new developments in therapy. After the introduction of antibody therapy around 2005, Her2neu-positive patients were increasingly treated with Trastuzumab, which positively influences the outcome. Some of the patients in the German validation-cohort received this kind of therapy, while others did not. Obviously, no clear trends for this subgroup can be deducted from analyzing such a heterogeneous sub-population.

The INFLUENCE-tool’s accuracy in the validation-cohort was poor according to the Hosmer–Lemeshow test. A fact, which must not be overrated. Of course, the *p* value is considerably lower than 0.05, which is commonly regarded as a reasonable threshold between good and poor accuracy. The discrepancy between predicted and observed values partly may be attributed to the large confidence intervals caused by the relatively small number of events in the German validation-cohort. However, even if this aspect is taken into account, one can see that observed and predicted values do not differ by mere coincidence. Actually, the INFLUENCE-algorithm systematically underestimates the actual risk in each of the risk-stratified quintiles. A reason for that might be that the LRR-rate in the German cohort is slightly—but not significantly—higher than in the Dutch modeling-cohort, while generally more adjuvant radio-, chemo, and endocrine therapies (which the INFLUENCE-nomogram associates with a lower LRR-risk) are performed. This could possibly reflect moderate differences in therapy perception between the two populations, which could be an interesting topic for further investigation.

For clinical use, accuracy is less important than discriminative ability, anyway. Health professionals seek to know whether their patients require intensified follow-up, because early detection of recurrence events is associated with superior outcomes (Lu et al. 2009; Sangen et al. 2013; Schneble et al. 2014). On the other hand, it is desirable to spare low-risk patients the psychological and the health care system as such the financial burden of overly intensive follow-up schemes (Puglisi et al. 2014). To develop personalized follow-up pathways, physicians most probably will use the INFLUENCE-nomogram together with some kind of cutoff. The ROC curve depicts sensitivity and specificity for every possible threshold which can be used with the INFLUENCE tool. The C-statistic/AUC, therefore, represents the discriminative ability of the algorithm. For the 5-year overall LRR-risk algorithm, the C-statistic/AUC was 0.71 in the Dutch modeling-cohort; almost the same value was obtained by the first external validation with another Dutch cohort from 2007 and 2008. With the German patients analyzed within this study, the C-statistic/AUC was even slightly larger (0.73); this indicates good external validity. The number 0.73 means that if a—from the statistical point of view—ideal threshold of 1.6% was chosen, more than 70% of the high risk and more than 65% of the low-risk patients would be classified correctly, which, if implemented in daily clinical practice, would be an important step towards personalized medicine. The prediction tool also turned out to be robust against differences in population features, as no decline in model performance could be seen in any of the age-, type of surgery-, and intrinsic biological subtype-stratified subgroup analyses, except Her2neu-positive patients. While this also may be attributed to the issues with this special subgroup discussed earlier, the re-evaluation of Her2neu as an independent predictor in the INFLUENCE-model should be considered. According to Witteveen et al. the implementation of Her2neu did not improve the performance of the INFLUENCE-nomogram and consequently was omitted. However, the algorithm is based on patients from 2003 to 2006, which, as previously mentioned, was a period of change, as far as Her2neu is concerned and nowadays it is believed to have considerable influence on the outcome of interest (Gamucci et al. 2013; McGuire et al. 2017).

Focusing on the time-dependent models, discrimination-ability shows a negative gradient. The C-statistic/AUC moderately decreases mildly from year 1 to 4; in year 5 it suddenly drops to 0.50, indicating that there is no discriminative ability left. Notably, this is not a random phenomenon to be observed only in the validation-cohort. Internal validation based on the modeling-cohort returned a C-statistic/AUC of 0.84 for the first year and constantly declined until year five to a C-statistic/AUC of 0.62. While it is not surprising that the model performance is better in the modeling than in the validation-cohort, it must be stated though, that the INFLUENCE-nomogram obviously has some difficulties in predicting late recurrence events. Maybe this issue could be solved by updating the INFLUENCE-tool on a more recent modeling-cohort and re-evaluating the set of influence variables, like proposed above. It has to be acknowledged though that the occurrence of LRR could be influenced by unknown confounders, which might impede substantial improvement of model performance (Meads et al. 2012).

## Conclusion

The INFLUENCE-nomogram can effectively assist health professionals in determining primarily cured breast cancer patients’ individualized risk for locoregional recurrence. Remarkably, its overall prognostic ability is close to equal when used either in the German validation or the Dutch modeling-cohort, thus underlining international generalizability. Future research should aim to incorporate other important influencing factors to further enhance time-dependent performance.
